# Cumulative carbon as a policy framework for achieving climate stabilization

**DOI:** 10.1098/rsta.2012.0064

**Published:** 2012-09-13

**Authors:** H. Damon Matthews, Susan Solomon, Raymond Pierrehumbert

**Affiliations:** 1 Department of Geography, Planning and Environment, Concordia University, 1455 de Maisonneuve Boulevard West, Montreal, Quebec, Canada H3G 1M8; 2 National Oceanographic and Atmospheric Administration, University of Colorado, Boulder, CO, USA; 3 University of Chicago, Chicago, IL, USA

**Keywords:** climate change, global warming, greenhouse gases, carbon dioxide, allowable emissions, climate stabilization

## Abstract

The primary objective of the United Nations Framework Convention on Climate Change is to stabilize greenhouse gas concentrations at a level that will avoid dangerous climate impacts. However, greenhouse gas concentration stabilization is an awkward framework within which to assess dangerous climate change on account of the significant lag between a given concentration level and the eventual equilibrium temperature change. By contrast, recent research has shown that global temperature change can be well described by a given cumulative carbon emissions budget. Here, we propose that cumulative carbon emissions represent an alternative framework that is applicable both as a tool for climate mitigation as well as for the assessment of potential climate impacts. We show first that both atmospheric CO_2_ concentration at a given year and the associated temperature change are generally associated with a unique cumulative carbon emissions budget that is largely independent of the emissions scenario. The rate of global temperature change can therefore be related to first order to the rate of increase of cumulative carbon emissions. However, transient warming over the next century will also be strongly affected by emissions of shorter lived forcing agents such as aerosols and methane. Non-CO_2_ emissions therefore contribute to uncertainty in the cumulative carbon budget associated with near-term temperature targets, and may suggest the need for a mitigation approach that considers separately short- and long-lived gas emissions. By contrast, long-term temperature change remains primarily associated with total cumulative carbon emissions owing to the much longer atmospheric residence time of CO_2_ relative to other major climate forcing agents.

## 1. Introduction: beyond greenhouse gas stabilization

For the past two decades, efforts to mitigate emissions of carbon dioxide and other greenhouse gases have centred around the goal of stabilizing atmospheric concentrations of these gases. This focus on atmospheric stabilization is historically rooted in the text of Article 2 of the United Nations Framework Convention on Climate Change (UNFCCC), in which is written:

The ultimate objective of this Convention … is to achieve … stabilization of greenhouse gas concentrations in the atmosphere at a level that would prevent dangerous anthropogenic interference with the climate system.

(UNFCCC [1])

Following this objective, a considerable body of literature has evolved to attempt to first quantify what could be considered to be a ‘dangerous’ level of climate change, and second to determine what levels of greenhouse gas stabilization are consistent with avoiding said climate changes [2–4].

There are several inherent difficulties with this approach, which have posed a considerable challenge to the progress of climate mitigation. Defining ‘dangerous’ levels of climate change is clearly a subjective exercise, which is difficult to incorporate in a compelling manner into the process of policy decision making. There has been a recent convergence in policy discussions towards a stated goal of limiting global warming to 2°C above pre-industrial temperatures [5]; while there is evidence that 2°C of global warming would avoid a number of important and potentially dangerous climate impacts (see [6] for a review of climate impacts associated with various levels of global temperature change), there is little by way of quantitative evidence that this represents a ‘safe’ policy target, and some climate scientists argue that 2°C would result in unacceptably severe impacts [7,8].

Even given some chosen target for global temperature change, however, it is extremely difficult within the paradigm of greenhouse gas *concentration* stabilization to define an appropriate policy target for greenhouse gas *emissions*. The reasons for this are threefold. First, the relationship between emissions and atmospheric concentrations is complex; achieving stabilized concentrations over time would clearly require large emissions reductions, but would also imply continued emissions at a changing level consistent with the level of natural sinks that evolve over time in a manner difficult to quantify [9,10]. Second, the relationship between greenhouse gas concentrations and temperature change is an elusive quantity that has preoccupied climate scientists for several decades. This ‘climate sensitivity’ has been estimated many times, but remains subject to at least a threefold probable uncertainty range which has not narrowed appreciably in 30 years of research [10]. Third, even given some known instantaneous temperature response to increased greenhouse gas concentrations, there is still a considerable lag between the point of atmospheric concentration stabilization and the eventual ‘equilibrium’ climate change. This lag results from the slow adjustment of the ocean and other slowly responding climate system components to the relatively rapidly increasing atmospheric forcing; consequently, the eventual temperature change associated with a given greenhouse gas stabilization level will not be fully realized for many centuries [11,12].

Taken together, these difficulties present no clear way to estimate by how much emissions must be decreased to avoid a given level of global temperature change, which may or may not be sufficient to avoid dangerous anthropogenic interference in the climate system. Many attempts have been made, and current policy discussions revolving around numbers like 50 per cent reductions in greenhouse gas emissions by 2050 followed by 80 per cent reductions at the end of the century (see UNFCCC [5], and analysis by Ramanathan & Xu [13]) do have some basis in climate science. For example, at least an 80 per cent reduction in carbon dioxide emissions is consistent with short-term concentration stabilization [6], and if enacted quickly enough as to stabilize concentrations below 450 ppm, this would give even odds of avoiding a long-term warming of 2°C [4]. But, there are many possible emissions pathways leading to an 80 per cent reduction, which could lead to considerably different atmospheric concentrations and temperature change, and what happens to emissions after an 80 per cent decrease is achieved will have an equally large bearing on the eventual climate change that occurs.

This preoccupation with atmospheric concentration stabilization—while cumbersome when applied to mitigation policy—is nevertheless consistent with the historical development of global climate models, which until recently have required the use of prescribed atmospheric greenhouse gas concentrations. However, the development of coupled climate–carbon models over the past decade has allowed for the investigation of the climate response to *emissions*, rather than *concentrations*, of carbon dioxide. With respect to carbon dioxide emissions—we will return to other gases later in the paper—this analysis has revealed several important conclusions.

— Global temperature change is approximately linearly related to a given amount of cumulative carbon dioxide emissions [14–17].
— This temperature change is independent of the specific pathway of carbon dioxide emissions, and depends only on the total carbon emitted over time [14–16].
— If carbon emissions are subsequently eliminated, global temperature changes will remain at near-constant levels for many centuries [10,18–22].
— The relationship between cumulative carbon and temperature change is remarkably constant and robust for cumulative emission up to about 2000 PgC, and on time scales from a decade to several centuries. This constancy holds within a given model (as well as for observations), although varies between models as a result of physical climate and carbon cycle uncertainty [14].

The policy implication of this body of literature is that a given level of cumulative carbon emissions can be uniquely associated with a given global temperature change. Consequently, the climate mitigation challenge can be simplified to the task of selecting an allowable cumulative emissions budget that is consistent with a given amount of global warming.

## 2. Cumulative carbon and global warming

The allowable carbon dioxide emissions pathway associated with a given CO_2_ stabilization scenario can be estimated with any coupled climate–carbon model when driven by prescribed CO_2_ concentrations; the requirement of global carbon conservation allows simulated carbon sink changes to be used to calculate the emissions profile that is required to drive the prescribed CO_2_ concentration changes [9]. Such simulations have been performed by several models [10], and have also been adopted as a primary methodology for simulations carried out in preparation for the upcoming Fifth Assessment Report of the Intergovernmental Panel on Climate Change [23].

**Figure 1.**
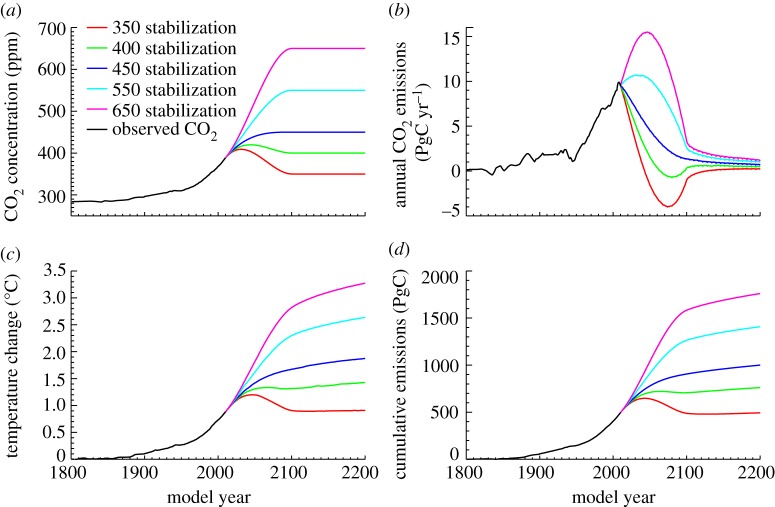
Climate and carbon cycle response to prescribed CO_2_ stabilization scenarios. (*a*) Prescribed atmospheric CO_2_ concentration (ppm). (*b*) Simulated allowable annual CO_2_ emissions (PgC yr^−1^), based on global carbon balance. (*c*) Simulated globally averaged temperature change relative to pre-industrial (°C). (*d*) Cumulative carbon emissions (PgC).

Figure 1 shows a series of such prescribed CO_2_ stabilization simulations carried out using an intermediate complexity coupled climate–carbon model.^1^ The scenarios shown here have atmospheric CO_2_ stabilizing at the year 2100 at levels between 350 and 650 ppm (figure 1*a*). Annual emissions (figure 1*b*) were diagnosed from annual changes in atmosphere, land and ocean carbon pools, and represent total CO_2_ emissions from both fossil fuels and land-use change. In all cases, allowable annual emissions decreased dramatically as global carbon sinks quickly saturated under stable atmospheric CO_2_ concentrations. Stabilizing CO_2_ below 400 ppm this century required prolonged periods of net negative emissions, although all stabilization targets allowed small amounts of continued emissions for several centuries after the point of atmospheric stabilization. Cumulative emissions (figure 1*d*) are equivalent to accumulated changes in simulated global carbon pools, and represent the total historical anthropogenic CO_2_ emitted to date in each simulation. Global temperature changes (figure 1*c*) responded to CO_2_ concentration such that there was substantial continued warming beyond the point of atmospheric concentration stabilization; this continued warming is consistent with the continued low-level emissions, leading to increasing cumulative carbon emissions over time which closely tracked the changes in global temperature.

**Figure 2.**
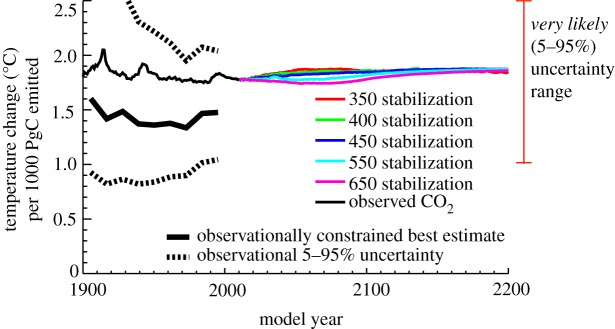
Simulated temperature change per 1000 PgC cumulative carbon emitted. Observational constraints for the twentieth century are given by the thick solid and dashed lines, as in Matthews *et al.* [14]. The *very likely* (5–95%) uncertainty range is indicated by the red error bar, based on a combination of estimates given by Matthews *et al.* [14] and Allen *et al.* [16].

This close association between cumulative emissions and global temperature change can be seen clearly in figure 2, which shows the temperature change per unit carbon emitted as a function of time in each model simulation. For all model simulations, temperatures increased by an approximately constant value of 1.8°C per 1000 PgC emitted; this linear response of global warming to cumulative emissions is a striking model property that is independent of both time and atmospheric CO_2_ concentration [14]. Matthews *et al.* [14] defined this as the *carbon–climate response* (CCR) and showed that a constant CCR is a robust feature of the current generation of coupled climate–carbon models—although with different models exhibiting different CCR values as a result of uncertainty in both climate and carbon cycle response to emissions. Further, they showed that the observational record (overlaid on figure 2 as the thick solid and dashed lines) showed a similar constancy of the temperature response to cumulative emissions, with a mean value of 1.5°C per 1000 PgC emitted, and a 5–95% range of 1 to 2.1°C/1000 PgC.

The importance of the temperature response to cumulative emissions was highlighted concurrently by Allen *et al.* [16], who used a simple climate model and a wide range of climate sensitivity values to calculate the *peak* temperature response to cumulative emissions of 1000 PgC. They defined this quantity as the *cumulative warming commitment* (CWC) and estimated a 5–95% CI of 1.3–3.9°C/1000 PgC. They further estimated the *instantaneous* temperature response to cumulative emissions (which is consistent with the CCR of [14]) to fall between 1.4 and 2.5°C/1000 PgC. Both Matthews *et al.* [14] and Allen *et al.* [16] concluded that the temperature response to cumulative emissions is remarkably constant over time and over a wide range of CO_2_ concentrations. Based on the uncertainty ranges estimated in these two studies, we have adopted a *very likely* (5–95%) uncertainty range of 1–2.5°C of global temperature increase per 1000 PgC of cumulative carbon emitted; this range is indicated by the red vertical bar to the right of figure 2.

This uncertainty in the temperature response to cumulative emissions stems from fundamental model uncertainties in: (i) the carbon cycle response to CO_2_ emissions (*carbon cycle sensitivity*); (ii) the climate response to changes in CO_2_ concentration (*climate sensitivity*); and (iii) the feedbacks between climate change and carbon sinks (*climate–carbon feedbacks*). When estimated from historical observations, the primary contributors to the total uncertainty are uncertainty in aerosol forcing (leading to uncertainty in climate sensitivity, or more specifically, the transient climate response) in addition to uncertainty in historical CO_2_ emissions from land-use change (which reflects uncertainty in the carbon cycle sensitivity) [14]. As with climate sensitivity, it is difficult to exclude the possibility of much higher values of the carbon–climate response, which would be consistent with either strong negative aerosol forcing or low emissions from land-use change in the historical record [14].

**Figure 3.**
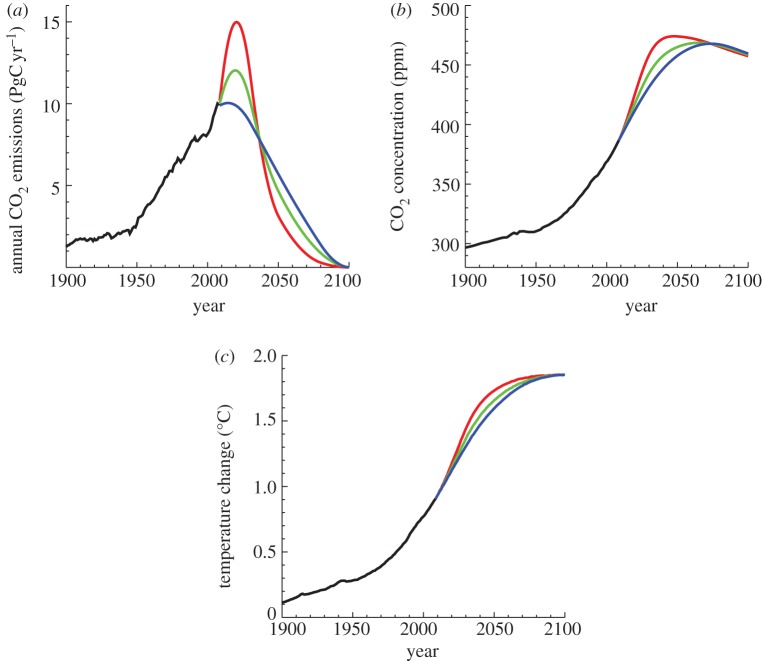
(*a*–*c*) Climate response to three emission scenarios, each with cumulative emissions equal to 1000 PgC. The transient rate of temperature change differs between scenarios, but both CO_2_ concentration (*b*) and temperature change (*c*) at the year 2100 are independent of scenario (*a*) and depend only on the cumulative emissions. Figure adapted from fig. 3.7 of Solomon *et al.* [6].

Despite uncertainty in its absolute value, the temperature response to cumulative emissions does not depend on the specific transient nature of a given emissions scenario. This scenario-independence is shown in figure 3, which shows the simulated model response to three CO_2_ emissions scenarios all of which have cumulative emissions of 1000 PgC (figure 3*a*). All three scenarios have CO_2_ emissions that peak and decline at rates between 1.5 and 4.5 per cent per year (relative to the peak emission value), and reach zero at the year 2100. Despite the different emissions rates over the twenty-first century, both year-2100 CO_2_ concentration (figure 3*b*) and temperature changes (figure 3*c*) are the same for all three simulations. While the transient changes in CO_2_ and global temperature do depend on the emission scenario, the final climate state depends only on the total cumulative emissions.

**Figure 4.**
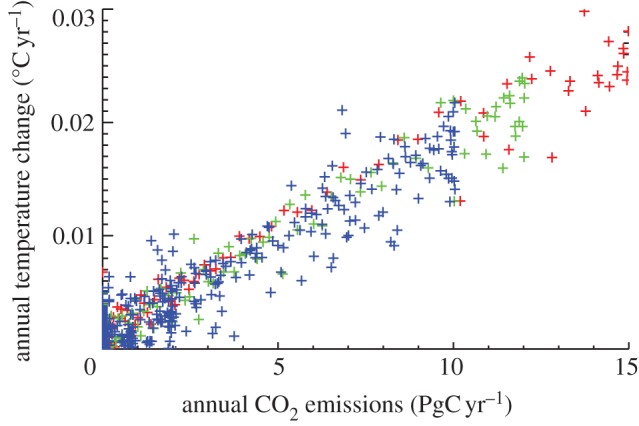
Response of annual temperature change to annual emissions for the simulations shown in figure 3. The *rate* of warming depends linearly on the rate of increase of cumulative emissions, whereas the total warming to date depends on the total cumulative emissions to date.

This dependence of transient climate change on the emissions scenario can be seen clearly in figure 4, which plots the annual temperature increase as a function of annual emissions for each of the three scenarios shown in figure 3. All points fall approximately on a line corresponding to 0.018°C per 10 PgC emitted (1.8°C per 1000 PgC)—which characterizes this model’s temperature response to cumulative emissions—with some variation from the line as a result of natural interannual variability in the model. The annual rate of temperature increase is therefore linearly related to the rate of increase of cumulative emissions; this relationship appears to be surprisingly constant over the range of emissions shown here. A key reason for this behaviour was emphasized by Caldeira & Kasting [28] who noted the compensation between increased retention of atmospheric carbon as emissions accumulate (linked to a slowdown in the ocean sink) and decreased radiative efficiency as stronger absorption bands saturate at higher CO_2_ concentrations. Whereas long-term temperature changes (and associated impacts) will be determined primarily by total cumulative emissions, short-term impacts that depend on the rate of climate warming will probably be more sensitive to the rate at which emissions increase or decrease over the next century.

In summary, cumulative carbon dioxide represents a powerful tool with which to assess the climate impacts of various levels of anthropogenic CO_2_ emissions. The following are robust conclusions that emerge from this framework of analysis.

— A given emission of carbon will lead to an approximately constant increment to global temperature, regardless of when or over how long this emission occurs.
— Uncertainty in the climate and carbon cycle response to emissions results in uncertainty in the temperature response to cumulative emissions.
— We can define a *very likely* (5–95%) range for the temperature response to cumulative emissions of 1–2.5°C per 1000 PgC emitted.
— At a given year, the global temperature change which occurs because of cumulative emissions to that date can be associated also with a unique atmospheric CO_2_ concentration (assuming reasonably similar CO_2_ emission scenarios).
— Stabilizing atmospheric CO_2_ concentrations at this level would allow continued emissions—and would correspondingly lead to continued climate warming—though if emissions are subsequently eliminated, CO_2_ concentrations would decrease over time and global temperatures would stabilize.
— The long-term temperature change depends only on cumulative emissions, and not on the rate of change of emissions over the next century.
— The transient rate of warming does depend on the emissions scenario, with faster increases in cumulative emissions leading to faster rates of warming over the next few decades.

## 3. Cumulative carbon, aerosols and other greenhouse gases

The use of cumulative carbon emissions provides a simple and versatile approach to the problem of climate change mitigation. This is particularly true for long-term temperature targets; because of the very long lifetime of anthropogenic CO_2_ in the atmosphere relative to most other climate-relevant gases [29,30], the climate warming signal will become increasingly CO_2_-dominated as we move into the latter half of this century and beyond. When considering nearer-term climate targets, however, and particularly if we are to restrict the overall rate of climate warming over the next several decades, it is not possible to ignore the effect of other greenhouse gases and aerosols.

**Figure 5.**
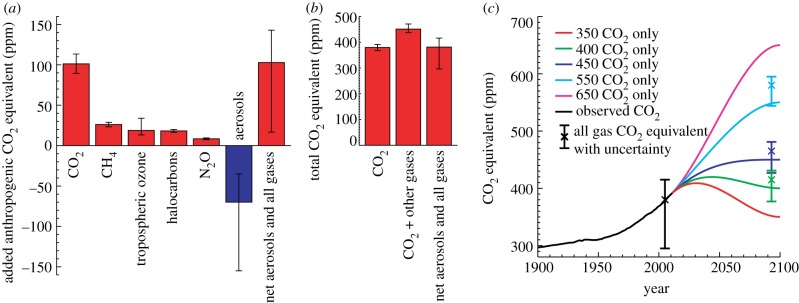
CO_2_-equivalent concentrations of other gases and aerosols. (*a*) Year-2005 CO_2_-equivalent of anthropogenic aerosols and all greenhouse gases, based on forcings given in Forster *et al.* [31]. Halocarbons (including chlorofluorocarbons, hydrocarbons, hydrofluorocarbons and perfluorocarbons) have been grouped, as have the direct and indirect effects of aerosols. (*b*) Year-2005 atmospheric CO_2_ concentration, the equivalent CO_2_ concentration including other greenhouse gases, and the equivalent CO_2_ including other greenhouse gases and aerosols. The balance of negative forcing from aerosols and positive forcing from other greenhouse gases shown here is such that the equivalent CO_2_ atmospheric concentration is very close to the current CO_2_-only atmospheric concentration. (*c*) Idealized CO_2_ concentration scenarios with the year-2005 CO_2_-equivalent range added, as well as year-2100 CO_2_-equivalent ranges for the 400, 450 and 550 stabilization scenarios. Year-2100 central values for CO_2_-equivalent (cross symbols) were taken from the RCP 2.6 (400 and 450 ppm) and RCP 4.5 (550 ppm) scenarios [32]. We assumed a year-2100 uncertainty range that was equivalent to that at year 2005, but decreased in proportion with the magnitude of the net aerosol forcing. (*a*) and (*b*) adapted from Solomon *et al.* [6, fig. 2.1].

The current balance of positive and negative forcings is such that the best estimate of the net anthropogenic forcing is very close to the forcing from CO_2_ alone. This can be seen in figure 5, which shows the estimate from Forster *et al.* [31] of the radiative forcing for 2005 from all radiatively active gases and aerosols, expressed in terms of equivalent CO_2_ concentration (figure 5*a*). When considering only positive forcings (CO_2_ and other greenhouse gases), the year-2005 CO_2_-equivalent concentration is close to 450 ppm (figure 5*b*). However, when negative forcings are also included, the CO_2_-equivalent concentration at 2005 is close to that of CO_2_ alone (figure 5*b*). This result has important policy implications: when aerosol forcing is included, it is clear that we have not yet reached the 450 ppm atmospheric CO_2_-equivalent concentration level that is generally associated with a long-term warming of 2°C above pre-industrial temperature.

Atmospheric lifetimes of non-CO_2_ greenhouse gases and aerosols vary considerably, from a few days (aerosols and tropospheric ozone) to a decade (methane) to a century and longer (nitrous oxide and halocarbons) [31]. If emissions of all aerosols and greenhouse gases (including CO_2_) were to be eliminated, one would expect an immediate warming (of uncertain magnitude, given the current large uncertainty associated with aerosol forcing), followed by a multi-decadal cooling owing to the decreases in atmospheric concentrations of methane and nitrous oxide [33–35]; on time scales of a century or so, the climate change signal would probably converge with that owing to CO_2_ alone [35]. This suggests that one approach to mitigation could be a two-basket method in which CO_2_ (and perhaps nitrous oxide and perfluorocarbons) would be dealt with in one basket to provide a multi-century constraint, whereas aerosols, methane and ozone precursors would be dealt with in another basket constraining shorter-term changes.

In a more realistic scenario where emissions of all gases change more slowly, it is less clear how the relative balance of positive and negative non-CO_2_ forcings would change over time. For some guidance on this question, we have drawn on the recent RCP scenarios [32], which provide information on changes in greenhouse gas and aerosol forcing over a range of mitigation scenarios. The scenarios RCP 2.6 and RCP 4.5 closely approximate our 400/450 and 550 stabilization scenarios, respectively. From these, we calculated the year-2100 CO_2_-equivalent with all anthropogenic forcings included in the same manner as for the year-2005 values shown in figure 5. In both RCP 2.6 and RCP 4.5, the fraction of radiative forcing due to CO_2_ alone increased relative to the total owing to all greenhouse gases (i.e. CO_2_ became more dominant among positive radiative forcing agents by the end of the century than it is today). However, aerosol forcing decreased more than non-CO_2_ forcing in both scenarios, leading to an increase in the CO_2_-equivalent concentrations at 2100. When applied to the scenarios here, CO_2_-equivalent concentrations, compared with the CO_2_-only concentrations, were 415 ppm versus 400, 465 ppm versus 450 and 580 ppm versus 550 (plotted as the cross symbols in figure 5*c*). In all cases, the uncertainty ranges (assumed to be equivalent to the uncertainty range at the year 2005, but decreased in proportion to the magnitude of the net aerosol forcing) overlapped the CO_2_-only concentrations.

From this analysis, we can conclude that the current close balance of positive (non-CO_2_ greenhouse gas) forcing and negative (aerosol) forcing is unlikely to persist throughout this century, though it is also unlikely to shift enough to result in dramatic deviations from the CO_2_-only results. For the range of RCP scenarios that we have analysed, there was some continued cancellation of these two sets of forcing, though the balance of forcings did move somewhat towards smaller aerosol relative to non-CO_2_ gas forcing. As a result, the CO_2_-equivalent concentration increased by 15–30 ppm relative to the CO_2_-only concentration. This clearly introduces some additional uncertainty into the climate response to cumulative carbon emissions, though in all cases, the CO_2_-only result fell within the uncertainty range of possible CO_2_-equivalent concentration when all forcings are included; consequently, the temperature response to cumulative carbon emissions remains a close approximation of the temperature response to cumulative carbon in combination with emissions of other greenhouse gases and aerosols.

## 4. Summary

Cumulative carbon represents a useful framework within which to assess the question of how to mitigate emissions so as to avoid dangerous anthropogenic climate impacts. The advantages of using cumulative carbon are clear. There appears to be a robust linear relationship between temperature change and cumulative carbon emissions, which greatly simplifies the very complex relationship between emissions, concentrations and temperature change. Furthermore, this framework allows an estimate of the instantaneous temperature response to cumulative emissions, which is approximately consistent with the long-term temperature in the absence of additional emissions; this avoids the difficulties inherent in the greenhouse gas stabilization framework associated with the large difference between transient and equivalent warming at a given atmospheric concentration. There remains a significant uncertainty associated with the magnitude of the temperature response to cumulative emissions, which emerges as a result of fundamental uncertainties in the carbon cycle response to emissions, the temperature response to changes in atmospheric concentrations, and the feedbacks between temperature change and carbon sinks. There is also additional uncertainty that reflects the relative balance of non-CO_2_ greenhouse gas and aerosol forcing over the next century, which is particularly relevant to near-term climate targets, and is of comparable magnitude to the climate and carbon cycle uncertainties.

**Figure 6.**
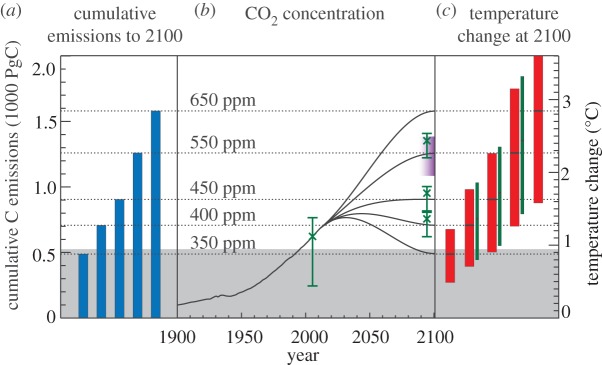
Summary figure showing the relationship between cumulative emissions, CO_2_ concentrations and temperature change. (*a*) Cumulative emission values, (*b*) CO_2_ scenarios and (*c*) the central value for the year-2100 temperature changes corresponding to the UVic ESCM model simulations as shown in figure 1. The red-bar temperature range represents the 5–95% uncertainty range for the temperature response to cumulative emissions [14,16]. In (*b*) the purple shaded region represents an estimate (for 550 CO_2_ scenario) of the uncertainty in the carbon cycle response to cumulative emissions, based on the C4MIP model simulations [36].^2^ Also shown in (*b*), for the year 2005 as well as for the year 2100 of the 400, 450 and 550 scenarios, are additional ranges corresponding to the CO_2_-equivalent values of CO_2_ plus non-CO_2_ greenhouse gases and aerosols (green cross symbols and uncertainty ranges, as plotted in figure 5*c*). Finally, for the scenarios where we included an estimate of the CO_2_-equivalent, we have included an additional range for the temperature response to cumulative emissions (thin green bars), shifted upward to match to the best estimate of the CO_2_-equivalent concentration for each of the 400, 450 and 550 ppm scenarios. The grey shaded region at the bottom of the plot shows total cumulative emissions to date, and the correspondingly inaccessible climate targets, assuming positive future cumulative emissions. Figure adapted from Solomon *et al.* [6, figs 3–8].

The cumulative carbon framework is summarized in figure 6. Read sequentially from left to right, this figure connects cumulative carbon emissions at the year 2100 with CO_2_ concentrations and temperature changes at that date. Uncertainties in temperature changes (red bars) reflect our estimate of the *very likely* (5–95%) range of temperature responses to the associated level of cumulative carbon emissions, based on carbon cycle and climate feedback uncertainties [6,14,16]. The uncertainty associated with the carbon cycle alone is indicated by the purple shaded region around the 550 ppm CO_2_ scenario at the year 2100, reflecting inter-model differences in the carbon cycle response to emissions and climate changes [36].^2^ The CO_2_-equivalent of all greenhouse gases and aerosols, along with the uncertainty on this estimate, is plotted on the CO_2_ concentration profiles with green cross symbols and error bars at year 2005, and at year 2100 for the three intermediate CO_2_ scenarios. For these scenarios (400, 450 and 550 ppm CO_2_ concentrations at 2100), we have also given a modified temperature response, which reflects the slight increase in the year-2100 CO_2_-equivalent concentration (relative to the CO_2_-only concentration) associated with a given level of cumulative carbon emissions (thin green vertical bars).

The shaded region at the bottom of figure 6 shows total historical cumulative emissions (about 530 PgC at the end of 2009 [37,38]). Areas within this shaded region represent CO_2_ and temperature targets that are likely inaccessible this century, assuming positive future emissions (though see Matthews [39] for a review of proposals for carbon cycle geoengineering aimed at achieving net negative CO_2_ emissions). Even when the negative forcing due to aerosols is considered, we have probably already exceeded the total cumulative emissions that are consistent with achieving CO_2_ concentrations of 350 ppm within this century. Similarly, we are fast approaching the level of cumulative emissions consistent with 1°C of global temperature change above pre-industrial (about 550 PgC), though there is a 5 per cent chance that this target could still be met with emissions up to about 1000 PgC. The most probable level of emissions for 2°C of global temperature change is about 1100 PgC, though it may be possible (5% likelihood) that 2°C will be reached with cumulative emissions as low as 800 PgC, or as high as 2000 PgC (figure 6; calculations based on a 5–95% range of 1–2.5°C per 1000 PgC).

According to this analysis, the year-2100 CO_2_ concentration most consistent with 2°C is 500 ppm, though this is predicated on the assumption of zero CO_2_ emissions after the year 2100. The values of temperature change shown here are only consistent with cumulative emissions over the entire span of time during which humans emit CO_2_; in order for the temperature changes shown here at the year 2100 to remain at that level further into the future, human emissions of CO_2_ must have reached zero by the year 2100.

Despite exceeding the cumulative emissions threshold for 350 ppm this century, as well as that for 1°C of global warming, we have almost certainly not yet reached a level of cumulative emissions that could result in 2°C of global temperature change. Meeting the stated international goal of 2°C over pre-industrial temperatures is clearly a difficult task that would require dramatic reductions and probably the eventual elimination of CO_2_ emissions this century. This may well be daunting, but it depends entirely on choices regarding future energy sources, and is far from an impossible objective.

## Footnotes

1 For the model simulations shown here, we have used the University of Victoria Earth-System Climate Model, a coupled global climate and carbon cycle model with interactive representations of three-dimensional ocean circulation, atmospheric energy and moisture balance, sea ice dynamics/thermodynamics, vegetation dynamics, and land, ocean and sedimentary carbon cycles [24–27].

2 We estimated the carbon cycle uncertainty based on the range of cumulative emissions at the time when atmospheric CO_2_ reached 550 ppm in the ‘coupled’ simulations from models participating in the C4MIP project; we discarded one model in this group as a clear outlier.
